# Quality indicators for the evaluation of end-of-life care in Germany – a retrospective cross-sectional analysis of statutory health insurance data

**DOI:** 10.1186/s12904-020-00679-x

**Published:** 2020-12-08

**Authors:** Katharina van Baal, Sophie Schrader, Nils Schneider, Birgitt Wiese, Jona Theodor Stahmeyer, Sveja Eberhard, Siegfried Geyer, Stephanie Stiel, Kambiz Afshar

**Affiliations:** 1 Institute for General Practice, Hannover Medical School, Carl-Neuberg-Straße 1, 30625 Hannover, Germany; 2 AOK Lower Saxony, Department for Health Services Research, Hildesheimer Str. 273, 30519 Hannover, Germany; 3 Medical Sociology Unit, Hannover Medical School, Carl-Neuberg-Straße 1, 30625 Hannover, Germany

**Keywords:** End-of-life care, Palliative care, Quality of health care, Health services research, Claims data

## Abstract

**Background:**

The provision and quality of end-of-life care (EoLC) in Germany is inconsistent. Therefore, an evaluation of current EoLC based on quality indicators is needed. This study aims to evaluate EoLC in Germany on the basis of quality indicators pertaining to curative overtreatment, palliative undertreatment and delayed palliative care (PC). Results were compared with previous findings.

**Methods:**

Data from a statutory health insurance provider (AOK Lower Saxony) pertaining to deceased members in the years 2016 and 2017 were used to evaluate EoLC. The main indicators were: chemotherapy for cancer patients in the last month of life, first-time percutaneous endoscopic gastrostomy (PEG) for patients with dementia in the last 3 months of life, number of hospitalisations and days spent in inpatient treatment in the last 6 months of life, and provision of generalist and specialist outpatient PC in the last year of life. Data were analysed descriptively.

**Results:**

Data for 64,275 deceased members (54.3% female; 35.1% cancer patients) were analysed. With respect to curative overtreatment, 10.4% of the deceased with cancer underwent chemotherapy in the last month and 0.9% with dementia had a new PEG insertion in the last 3 months of life. The mean number of hospitalisations and inpatient treatment days per deceased member was 1.6 and 16.5, respectively, in the last 6 months of life. Concerning palliative undertreatment, generalist outpatient PC was provided for 28.0% and specialist outpatient PC was provided for 9.0% of the deceased. Regarding indicators for delayed PC, the median onset of generalist and specialist outpatient PC was 47.0 and 24.0 days before death, respectively.

**Conclusion:**

Compared to data from 2010 to 2014, the data analysed in the present study suggest an ongoing curative overtreatment in terms of chemotherapy and hospitalisation, a reduction in new PEG insertions and an increase in specialist PC. The number of patients receiving generalist PC remained low, with delayed onset. Greater awareness of generalist PC and the early integration of PC are recommended.

**Trial registration:**

The study was registered in the German Clinical Trials Register (DRKS00015108; 22 January 2019).

**Supplementary Information:**

The online version contains supplementary material available at 10.1186/s12904-020-00679-x.

## Background

In 2018, approximately 955,000 people died in Germany [1]. It is assumed that roughly 75% of all people at the end of life require palliative care (PC) [2–4]. Given estimates that the number of patients with PC needs will increase in the coming decades, health care systems are expected to face significant challenges [5]. PC is generally provided for patients with oncologic diseases, while patients with non-oncologic chronic progressive diseases often receive PC at only a late stage in their disease trajectory [6–8]. Therefore, the World Health Organization has emphasised the importance of improving access to PC, especially for patients with non-oncologic diseases [9].

In Germany, outpatient PC includes both generalist and specialist PC. Generalist PC for patients in the community is mostly initiated and provided by primary care professionals (most frequently general practitioners). It is intended for patients at an early stage in their disease trajectory with overall low symptom intensity [10]. Since 2013, generalist outpatient PC in Germany has been available for statutory health insurance billing [11]. In contrast, specialist outpatient PC is typically provided by interdisciplinary teams comprised of trained specialists in PC for patients with complex problems and symptoms. Specialist outpatient PC is governed by the 2007 German Act to Strengthen Competition in statutory health insurance, and can be prescribed by both outpatient and inpatient physicians [12].

In 2015, Germany introduced legislation to improve hospice and PC (HPG) [13, 14]. Specifically, the new act aimed at developing generalist outpatient PC and regulating specialist outpatient PC [13]. This act states that *“palliative care is part of health care”* (e.g. §27 social security statutes (SGB) V) and comprises concrete implications for clinicians who provide specialist outpatient PC (e.g. §132d SGB V) [13, 14]. It promotes new forms of cooperation between interdisciplinary specialist outpatient PC teams and aims to improve care especially in regions with poor access to specialist PC services [13, 15].

It is unclear, however, whether the resulting structural and political developments led to significant changes in the provision and quality of end-of-life care (EoLC).

Radbruch et al. evaluated EoLC in Germany in the years 2010 to 2014 according to three categories of quality indicators [7]:

1. curative overtreatment (e. g. chemotherapy in the last month of life);
2. palliative undertreatment (e. g. generalist outpatient PC in the last year of life); and
3. delayed PC (e. g. onset of specialist outpatient PC before death).

Other relevant analyses have focused on regional disparities, as well as the structures and utilisation of PC throughout Germany [7, 15]. Radbruch et al. identified different PC patterns across the federal states, but an overall focus on curative care, over and above caring and accompanying approaches. The researchers recognised overtreatment with curative approaches at the end of life in most German regions, even when medical indications of the utility of such approaches were lacking [7]. In this context, it is reasonable to assume that the potential damage of curative treatment may outweigh the benefits [16]. At the same time, palliative treatment approaches may fall short, indicating palliative undertreatment. Future actions recommended by Radbruch et al. involved improving access to PC and raising awareness of the need for PC amongst health care professionals [7].

The aim of the present study was to evaluate current EoLC on the basis of quality indicators similar to those used by Radbruch et al. [7] Statutory health insurance data from the years 2016 and 2017 pertaining to deceased members’ last year of life were analysed and compared with Radbruch et al.’s findings from 2010 to 2014 [7]. Specifically, the following questions were addressed:

1. In 2016 and 2017, what was the quality of EoLC in Lower Saxony, Germany, on the basis of curative overtreatment, palliative undertreatment and delayed provision of PC?
2. To what extent do the data from 2016 and 2017 differ from the data from 2010 to 2014?

For the comparison with 2010 to 2014 data, we assumed the following hypotheses:

1. Curative overtreatment: the 2016 and 2017 data would show a reduction in curative overtreatment and aggressive treatment at the end of life.
2. Palliative undertreatment: the 2016 and 2017 data would demonstrate that specialist and especially generalist PC were provided more often, indicating an increased awareness for PC needs amongst health care workers.
3. Delayed provision of PC: The 2016 and 2017 data would show that both generalist and specialist PC were initiated early in patients’ disease trajectory.

## Methods

### Study design

A retrospective secondary analysis of statutory health insurance data was performed through a cross-sectional study following the RECORD Statement (Reporting of studies Conducted using Observational Routinely-collected Data) [17]. The study was part of the research project entitled “Optimal care at the end of life” (OPAL) [18], which aims at improving EoLC in selected rural regions in Lower Saxony, Germany.

### Study population

AOK (Allgemeine Ortskrankenkasse) is one of the largest statutory health insurance providers in Germany. With more than 2.8 million insured members in Lower Saxony, AOK holds reliable data on approximately 36% of state residents [19]. Specifically, AOK collects demographic and sociodemographic data, as well as outpatient and inpatient diagnoses and treatments, for accounting purposes. For the present study, we used data pertaining to AOK Lower Saxony (AOK-N) members who died in 2016 or 2017, as these were the most recent available data. In this study, we included insured members with residence in Lower Saxony who needed to be at least 18 years old at the time of death and to be continuously insured in the year of death and the preceding calendar year. An additional inclusion criterion was the presence of a valid diagnosis for at least one chronic progressive oncologic or non-oncologic disease (Table 1) in the last year of life. We accepted diagnoses in the outpatient setting as valid, if the associated codes in the *International Statistical Classification of Diseases and Related Health Problems – 10th Revision* (ICD-10) were documented in at least two of the five quarters preceding death (i.e., the quarter of death and the four preceding quarters). For inpatient diagnoses, a single diagnosis was considered sufficient for inclusion. Non-chronic conditions and suspected diagnoses were excluded [20, 21].

**Table 1:** Diagnosis groups and ICD-10 code list

ICD-10 codes	Diagnosis group
B20-B24	HIV/AIDS
C00-C97	Malignant neoplasms
I25, I27, I28, I31, I32, I38, I42-I52	Heart diseases
I60-I64, I67-I69	Cerebrovascular diseases
N18, N28	Renal diseases
K70-K77	Liver diseases
J41-J45, J47, J96, E84	Respiratory diseases
G10, G12, G20, G23, G35, G71	Neurodegenerative diseases
F00, F01, F03, G30, R54	Dementia, Alzheimer’s, senility/frailty

Diagnoses of interest were predefined according to the ICD-10 and based on data from Murtagh et al. [22] and Rosenwax et al. [2] The ICD-10 code list was adjusted by an interdisciplinary expert council comprised of two physicians (a specialist and a trainee for family medicine and PC), a nursing scientist, a sociologist, a health scientist and a physiotherapist. Acute diagnoses, risk factors, conditions leading to chronic diseases without an immediate need for PC and diseases that do not require PC (from a clinical perspective) were excluded. In contrast to Radbruch et al., we have focused our analyses on chronic diseases and diseases that potentially cause PC needs.

### Outcomes

Data from the deceased were analysed on the basis of approved quality indicators for the evaluation of EoLC, as described by Radbruch et al. [7] The published findings of Radbruch et al. on these quality indicators were used as a baseline from which to compare the EoLC findings of the present study [7]. Most of the relevant quality indicators are well-established and described in the international literature [23, 24]. Of note, AOK-N was unable to provide complete data on chemotherapy treatments for deceased members in 2016, which is why the results for this indicator refer only to 2017. Table 2 shows the quality indicators examined in the present study.

**Table 2:** Quality indicators for EoLC [7]

Quality indicator	Time reference
Curative overtreatment	
Chemotherapy (cancer patients)	Last month of life (30 days)
New insertion of a PEG tube (dementia patients)	Last three months of life (90 days)
Hospitalisations and days spent in inpatient treatment (at least one overnight stay)	Last six months of life (180 days)
Palliative undertreatment	
Generalist outpatient PC	Last year of life (365 days)
Specialist outpatient PC	Last year of life (365 days)
Delayed PC	
Onset of generalist outpatient PC prior to death (days)	–
Onset of specialist outpatient PC prior to death (days)	–
Initiation of specialist outpatient PC	Last three days of life

*PC* Palliative care, *PEG* Percutaneous endoscopic gastrostomy.

### Data analysis

Data were analysed descriptively using the software IBM Statistical Package for Social Sciences version 26 (SPSS Inc., Chicago, IL/USA).

### Data protection

The present study followed the data security procedure described in the study protocol of the main research project (OPAL) [18]. AOK-N edited and anonymised data from the deceased before transferring them to the study team. Both project partners discussed and agreed on the anonymisation procedure in advance. As an example of this procedure, age groups were defined as broadly as possible, to ensure data security and to prevent the backtracking of individuals. All data were saved and stored on a secure and password-protected institutional server. Data processing was conducted by the study team, exclusively.

## Results

### Description of the study sample

**Fig. 1:**
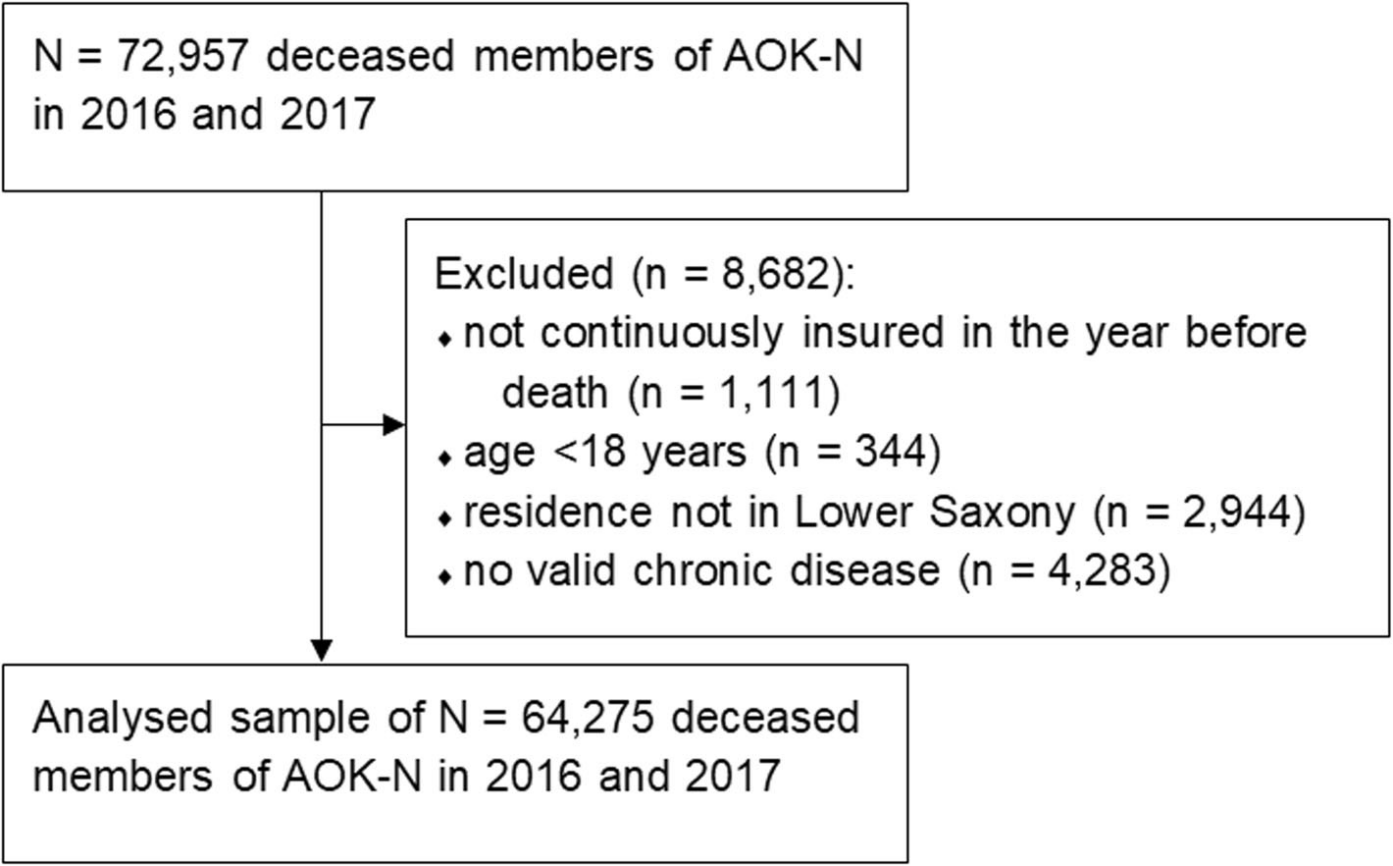
Flow chart of the inclusion and exclusion of deceased members of AOK-N

The present analysis used data pertaining to 64,275 deceased members (2016: 32,442; 2017: 31,833). The mean age of death was 80.0 years (SD 11.9): 82.9 years (SD 11.2) for females and 76.6 years (SD 11.9) for males. Figure 1 shows the inclusion and exclusion decisions for the deceased members. The final sample contained a slightly higher proportion of women. Table 3 presents the demographic characteristics of the study population.

**Table 3:** Demographic characteristics of the deceased

Characteristics	n	%
Sex		
Female	34,878	54.3
Male	29,397	45.7
Age groups		
18–50	1363	2.1
51–60	3761	5.9
61–70	7234	11.3
71–80	15,620	24.3
81–90	25,017	38.9
> 90	11,280	17.5
Disease groups^a^		
HIV/AIDS	61	0.1
Malignant neoplasms	22,567	35.1
Heart diseases	48,009	74.7
Cerebrovascular diseases	19,014	29.6
Renal diseases	25,884	40.3
Liver diseases	10,370	16.1
Respiratory diseases	30,138	46.9
Neurodegenerative diseases	4216	6.6
Dementia, Alzheimer’s, senility/frailty	32,207	50.1
Death in hospital		
Yes	30,174	46.9
No	34,101	53.1

^a^at least one valid diagnosis in this group

### EoLC quality indicators

The descriptive analyses of the evaluation of EoLC on the basis of quality indicators are presented in Table 4.

**Table 4:** EoLC quality indicators

Indicator	n	%
Chemotherapy (cancer patients; *N* = 10,758)		
Yes	1118	10.4
No	9640	89.6
New PEG tube insertion (dementia patients; *N* = 32,207)		
Yes	286	0.9
No	31,921	99.1
Number of hospitalisations^a^ (*N* = 64,275)		
0	14,422	22.4
1	21,599	33.6
2–3	21,350	33.2
≥ 4	6904	10.7
Number of treatment days^a^ (*N* = 49,853)		
1–3	6074	12.2
4–7	7316	14.7
8–14	11,168	22.4
15–30	14,147	28.4
31–60	8465	17.0
61–100	2126	4.3
≥ 101	557	1.1
Generalist outpatient PC (*N* = 64,275)		
Yes	18,021	28.0
No	46,254	72.0
Onset of generalist outpatient PC before death^a^ (days; *N* = 18,021)		
0–3	2062	11.4
4–10	2201	12.2
11–20	1869	10.4
21–30	1313	7.3
31–60	2514	14.0
61–120	2353	13.1
121–240	2256	12.5
≥ 241	3453	19.2
Specialist outpatient PC (*N* = 64,275)		
Yes	5753	9.0
No	58,522	91.0
First specialist outpatient PC in the last 3 days of life (*N* = 5753)		
Yes	761	13.2
No	4992	86.8
Onset of specialist outpatient PC before death (days; *N* = 5753)		
0–3	839	14.6
4–10	1013	17.6
11–20	819	14.2
21–30	551	9.6
31–60	935	16.3
61–120	818	14.2
121–240	528	9.2
≥ 241	250	4.3

*PC* Palliative care, *PEG* Percutaneous endoscopic gastrostomy; ^a^minor differences due to rounding.

### Curative overtreatment

In total, 10.4% of the deceased members with cancer (in 2017, only) received chemotherapy in the last month of life. The incidence of chemotherapy decreases with age (18–50 years old: 23.2%; 51–60: 16.9%; 61–70: 12.2%; 71–80: 8.6%; 81–90: 3.2%; > 90: 0.5%). Furthermore, 0.9% of all deceased members with a dementia diagnosis had a percutaneous endoscopic gastrostomy (PEG) tube inserted for the first time in the last 3 months of life (Table *4*). More than three-quarters of the deceased had at least one hospitalisation in the last 6 months of life (Table *4*), while the mean number of hospitalisations per deceased member was 1.6 (SD 1.5). Simultaneously, the mean number of days spent in inpatient treatment was 16.5 (SD 20.8).

### Palliative undertreatment

In the last year of life, 28.0% of the deceased received generalist outpatient PC. Specialist outpatient PC was provided for 9.0% of the deceased (Table *4*).

### Delayed provision of PC

For 41.3% of the deceased, generalist outpatient PC was provided for the first time within the last 30 days of life. The mean onset of generalist outpatient PC was 104.6 days before death (SD 118.4), with a median of 47.0 days (interquartile range (IQR) 12.0–180.0).

Moreover, 13.2% of the deceased with specialist outpatient PC received this service for the first time in the last 3 days of life, and 56.0% received it for the first time in the last 30 days of life. The mean onset of specialist outpatient PC was 53.9 days before death (SD 73.1), with a median of 24.0 days (IQR 7.0–68.0).

## Discussion

The main findings of this study were: (1) an increase and slightly earlier initiation of specialist outpatient PC, (2) a constant frequency and ongoing late initiation of generalist outpatient PC, (3) a reduction in the number of new PEG insertions in the last 3 months of life for patients with dementia and (4) a lower number of inpatient treatment days though an unchanged number of hospitalisations. In the following, we will discuss these results in comparison with earlier results and particularly with the published findings of Radbruch et al., who investigated EoLC on the basis of similar quality indicators in Germany for the years 2010 to 2014 (supplementary Table S1) [7].

### Curative overtreatment

Our results regarding curative overtreatment present a mixed picture. The lower number of dementia patients with a new PEG insertion in the last 3 months of life can be interpreted as a step in the right direction. Nonetheless, the relatively high proportion of cancer patients receiving chemotherapy in the last month of life and the high number of overnight hospital stays suggest an ongoing pattern of curative overtreatment.

The number of new PEG tube insertions in the last 3 months of life in 2016 and 2017 was considerably lower than that found by Radbruch et al. (2010 to 2014: 2.5%) [7]. It has been demonstrated that tube feeding does not improve clinically important outcomes, and it should therefore not be used, especially for patients with dementia [25]. For these patients, van der Steen et al. recommend intensified hand feeding, rather than permanent enteral tube nutrition [26]. Furthermore, insertion of a PEG tube is often perceived as burdensome by the general public and some health care professionals [7]. The decrease in new PEG insertions found in the present study may indicate an increase in the use of intensified hand feeding, as well as a higher awareness amongst health care professionals of the clinical limitations of PEG tubes at the end of life. The decrease may have also been affected by recent political initiatives and legal regulations in Germany, which may have improved PC awareness amongst health care professionals. Additionally, the new legislation to improve PC [14] may have encouraged the realisation of advance care planning concepts [13]. Therefore, undesirable overtreatments such as PEG tube insertions might continue to be reduced, especially within nursing homes, where they are often used for dementia patients at the end of life [13].

Compared to the findings of Radbruch et al. [7], the present results showed a slight increase in the number of cancer patients receiving chemotherapy in the last month of life (2010 to 2014: 9.6%). It would be incorrect to assume that all chemotherapy administered in the last month of life is inappropriate, as such treatment may be reasonable for patients with a fast disease progression or when aimed at improving quality of life [7]. However, exceedingly aggressive treatments (e.g. chemotherapy) at the end of life are indicative of poor EoLC, and they may negatively impact on patients’ quality of life [24, 27, 28]. There are many reasons why chemotherapy may still be administered in the last month of life. Clinicians may overestimate the prognosis, applying inappropriate treatment and delaying PC [29, 30]. Decisions on treatment intensity at the end of life may also be influenced by patient preferences. However, most patients at an older age prefer palliative treatment over life-extension treatment [31]. Early end-of-life conversations about patients’ preferences and the timely initiation of PC may reduce the administration of chemotherapy, thereby improving patients’ quality of life and care [32, 33]. Further PC education amongst health care professionals may encourage the provision of PC and reduce curative overtreatment [34, 35].

Compared to the results of Radbruch et al. [7], the present findings showed a consistent mean number of hospitalisations in the last 6 months of life, but a slightly lower (by approximately 2 days) number of inpatient treatment days (2010 to 2014: 1.7/18.6). The hospitalisation of patients with PC needs can sometimes be useful. However, hospital admissions with no medical indication may be deemed aggressive and burdensome by patients with PC needs at the end of life [7, 36, 37]. In Germany, the number of days spent in inpatient treatment has decreased over recent years, mainly due to changes in the health care system [38, 39]. Therefore, the lower number of hospital treatment days found in the present study cannot necessarily be interpreted as an indication of a reduction in curative overtreatment. Indeed, hospital admissions and treatment days may be influenced by a variety of factors, including the tendency for patients to feel safe in a hospital and general patient characteristics (e.g. age, ethnicity) [40]. Furthermore, it is often difficult for physicians to determine the clinical need for hospital admissions [37], and this may be one reason for the overall high number of hospitalisations at the end of life. Training in caregiving for terminally ill patients might improve this situation. Also, changes in the health care system to expand outpatient care alternatives for critically ill patients may be useful [37]. Further studies should investigate the effects of various approaches to reduce unnecessary end-of-life hospital admissions, such as PC training for ambulance staff [41].

### Palliative undertreatment

Compared to the results of Radbruch et al., the present findings showed a reduction in palliative undertreatment for specialist outpatient PC, but a consistent level of generalist outpatient PC, and therefore ongoing palliative undertreatment [7].

This consistency (2014: 28.0%) is highly remarkable, given the introduction of billing codes for generalist outpatient PC in Germany in 2013, which was expected to significantly increase the provision of this service. In fact, recent legal changes in Germany appear to have failed to achieve their intended goals, for a variety of reasons. As recently described, generalist outpatient PC requires great effort, especially from general practitioners [42]. Thus, there may be a need for further legislation around health care structures and financial models [42, 43]. A reform of payment models and funding approaches may improve widely access to PC, ensure best practice and prevent inverted incentives [43, 44]. Additionally, general practice has taken on greater importance in recent years and, in line with this, the requirements and qualifications for general practitioners have become increasingly complex [45]. However, the increased demand for primary care services has not been accompanied by an equivalent growth in the workforce; thus, time restraints on general practitioners might reduce their quality of care and lower their job satisfaction [46, 47]. Overall, time-consuming bureaucratic procedures, personal commitments and inadequate qualifications may prevent general practitioners from timely initiating PC, and this needs to be addressed [35]. Nevertheless, the present results do not enable any conclusions to be drawn relating to the daily care routines of general practitioners, since only billed health care services were included in the analysis.

Finally, the present results indicated a considerable increase in specialist outpatient PC relative to Radbruch et al.’s findings (2010 to 2014: 5.3%) [7]. It has been estimated that, in recent years, approximately 10% of the deceased required specialist outpatient PC prior to their death [48], but were unable to access this service [7, 49]. One important reason for the increase in specialist outpatient PC found in the present study might be the wider availability of specialist outpatient PC following its regional implementation in the community [50, 51]. In fact, the present findings indicate that the capacity for specialist outpatient PC has increased and it can be assumed that the estimated population need for specialist PC is met. Presumably, the legal changes and initiatives to raise awareness for palliative needs have contributed to this increase since 2014. This shows that structural and legal changes can be an important driver for further development in health care systems. Existing structures need to be improved and expanded from top to down and cannot solely develop on regional level. Differences in regional structures and processes of the specialist outpatient PC teams might play a key role. There might also be certain regional disparities between the counties in Lower Saxony. While the potential population need might be met in some regions, it is potentially missed in others. However, our data cannot distinguish whether those patients with the greatest needs are actually the ones provided with specialist outpatient PC.

### Delayed PC

The present findings underlined the ongoing late initiation of generalist outpatient PC. In contrast, specialist PC was initiated slightly earlier, relative to the findings of Radbruch et al. (2010 to 2014: median of 22.0 days) [7]. While the slightly earlier initiation of specialist outpatient PC found in the present study may suggest a step in the right direction, the number of days between the onset of this treatment and death – especially with regards to generalist outpatient PC – indicates an unchanged focus on the last months of life.

It is well known that the early initiation of PC improves many important outcomes, such as quality of life and the burden of symptoms [3, 52–54]. PC must not be reserved solely for patients whose life-prolonging treatment options have been exhausted; rather, it should be considered shortly after diagnosis [55]. Many physicians find it difficult to determine the appropriate time to initiate PC in the disease progression [56, 57]. Prognostic uncertainties form a major barrier for the early identification of patients with PC needs, and the estimation of disease progression is especially difficult for patients with non-oncologic diseases [6, 58]. Internationally, there are several instruments that support the identification of patients with potential PC needs [59, 60]. One such instrument is the Supportive and Palliative Care Indicators Tool (SPICT-DE), which is available for use in the German context [61, 62]. Its application in primary care is currently being evaluated [18]. Nonetheless, identification instruments such as the SPICT-DE are not implemented widely and consistently throughout Germany [63]. For this reason, further PC training for physicians and other health care professionals might represent an important step in supporting the identification of patients with potential PC needs and promoting the early initiation of PC [64–66].

### Methodological strengths and limitations

AOK-N is the largest statutory health insurance provider in Lower Saxony [19], and thus a reliable data source for the present analysis. The population of AOK-N members is comparable to the general population in Germany and Lower Saxony, regarding gender and age [67]. However, differences exist with respect to education and occupation, which is why lower socioeconomic groups may have been overrepresented in the current study [67]. To counteract this possible bias, the present study did not focus on socioeconomic differences between groups. Furthermore, the results were based on a large sample of AOK-N members who died in 2016 or 2017, enabling robust analyses to be conducted.

One difficulty with all secondary analyses of health insurance data pertains to billing purpose. In the present study, conclusions regarding PC timing may have been unreliable in some cases. Data on inpatient stays and outpatient services (e.g. generalist outpatient PC) were highly reliable, as they contained the dates of service provision. However, data on specialist outpatient PC only contained the date of prescription, while the actual treatment by a specialised PC team may have been delayed. Furthermore, specialist outpatient PC may have been initially prescribed by hospital doctors, and such prescriptions were not observable in the current dataset. Nonetheless, all follow-up prescriptions in the outpatient sector were observed. Finally, the use of routinely collected data involves low expenditure for data collection and can be highly beneficial to reflect the care situation [68]. However, it has to be taken into account that the actual care situation cannot be completely represented by routinely collected data.

### Content-related strengths and limitations

Although the data enabled us to evaluate the quality of EoLC on the basis of documented procedures of care, they did not allow us to analyse potential consequences of the analysed indicators, such as the effects on patients’ quality of life.

Further limitations pertain to diagnostic accuracy. Criteria for the validity of diagnoses cannot prove whether the diagnoses were correct and if patients were treated accurately [20]. Particularly in the outpatient sector, ICD-10 codes are often used imprecisely, due to variations in coding methods [69]. Data on diagnoses can be affected by an individual coder as well as by financial incentives in the German health care system. Additionally, statutory health insurance data does not record cause of death.

While the comparison with the results of Radbruch et al. [7] was reasonable to contextualise our data, considerable differences existed between the study samples. In contrast to Radbruch et al., the present study predefined chronic diseases with potential PC needs. Nonetheless, the utilisation of criteria for the validity of diagnoses was an important strength of our study. Only data from patients with valid chronic diagnoses were included in the analysis. Furthermore, the ICD-10 code list was based on the current literature [2, 22] and compiled by an interdisciplinary panel of experts.

## Conclusions

In addition to finding a decrease in new PEG insertions and an increase in specialist outpatient PC at the end of life, the present study also showed an ongoing pattern of curative overtreatment, palliative undertreatment and delayed provision of generalist PC. Particularly with regards to generalist outpatient PC, the findings suggest room for improvement. The legal amendments led to crucial changes in the provision of EoLC in Germany, but the need especially for generalist outpatient PC is still unmet.

In conclusion, there is a need for early end-of-life discussions, more timely initiation of PC and further PC training among health care providers. With regards to this latter point, increased awareness of PC needs is especially necessary in primary care. The wide and consistent use of standardised instruments to systematically identify patients with potential PC needs may improve EoLC by supporting the transition from curative overtreatment and palliative undertreatment to early integrated PC. Additionally, there is a need for further legislation concerning health care structures and financial models including strategies to strengthen the role of general practitioners in providing EoLC. Existing structures need to be expanded. Our results are based on the most recent available data and form the groundwork for a regular evaluation of EoLC.

## Supplementary Information

**Additional file 1: Table S1.** Comparison of EoLC quality indicators in Lower Saxony.

## Data Availability

Data from the statutory health insurance provider AOK-N are not publicly available due to data privacy protection regulations.

## Abbreviations

AOK-N: Statutory local health care fund in Lower Saxony
EoLC: End-of-life care
HPG: Act to improve hospice and palliative care
ICD-10: *International statistical classification of diseases and related health problems – 10th revision*
IQR: Interquartile range
OPAL: Optimal care at the end of life
PC: Palliative care
PEG: Percutaneous endoscopic gastrostomy
RECORD: Reporting of studies Conducted using Observational Routinely-collected Data
SD: Standard deviation
SGB: Social security statute
SPICT-DE: German version of the Supportive and Palliative Care Indicators Tool
