# Supplementary material for: Quality indicators for the evaluation of end-of-life care in Germany – a retrospective cross-sectional analysis of statutory health insurance data
Source: BMC Palliat Care. 2020 Dec 8;19:187. doi: 10.1186/s12904-020-00679-x (PMC7724721; doi:10.1186/s12904-020-00679-x)
Supplement: Supplementary file 1 — Additional file 1: Table S1. Comparison of EoLC quality indicators in Lower Saxony. [file 12904_2020_679_MOESM1_ESM.docx]

**Supplementary Table S1.** Comparison of EoLC quality indicators in Lower Saxony

| Indicator | | 2010-2014 [7] | 2016-2017 |
| --- | --- | --- | --- |
| Chemotherapy  (cancer patients; %) | Yes | 9.6 | 10.4 |
|  | No | 90.4 | 89.6 |
| New PEG tube insertion (dementia patients; %) | Yes | 2.5 | 0.9 |
|  | No | 97.5 | 99.1 |
| Number of hospitalisations  (mean) | | 1.7* | 1.6 |
| Number of treatment days  (mean) | | 18.6* | 16.5 |
| Generalist outpatient PC | Yes | 28.0 | 28.0 |
|  | No | 72.0 | 72.0 |
| Onset of generalist outpatient  PC before death  (days; median) | | -** | 47.0 |
| Specialist outpatient PC | Yes | 5.3 | 9.0 |
|  | No | 94.7 | 91.0 |
| First specialist outpatient PC in the last 3 days of life | Yes | 13.8 | 13.2 |
|  | No | 86.2 | 86.8 |
| Onset of specialist outpatient  PC before death  (days; median) | | 22.0 | 24.0 |

PC = palliative care, PEG = percutaneous endoscopic gastrostomy, *nationwide value, **not assessed in 2010-2014. Data on 2010-2014 refer to Radbruch et al. [7]
